# Safety of nanoparticle albumin-bound paclitaxel administered to breast cancer patients with clinical contraindications to paclitaxel or docetaxel: Four case reports

**DOI:** 10.3892/ol.2013.1471

**Published:** 2013-07-17

**Authors:** KOSEI KIMURA, SATORU TANAKA, MITSUHIKO IWAMOTO, HIROYA FUJIOKA, YUKO TAKAHASHI, NAYUKO SATO, RISA TERASAWA, TOMO TOMINAGA, AYANA IKARI, KAZUHISA UCHIYAMA

**Affiliations:** Section of Breast and Endocrine Surgery, Department of General and Gastroenterological Surgery, Osaka Medical College, Takatsuki, Osaka 569-8686, Japan

**Keywords:** breast cancer, nab-paclitaxel, hypersensitivity reactions

## Abstract

Taxanes, including paclitaxel (PTX) and docetaxel (DOC), are poorly soluble in water due to their hydrophobic properties and thus, require solvents. However, use of these solvents has been associated with toxic responses, including a hypersensitivity reaction (HSR). Nanoparticle albumin-bound paclitaxel (nab-PTX) is a novel formulation of PTX, which allows reconstitution of the agent with a saline solution instead of solvents and administration without premedication for HSRs. The current study reports the safe administration of nab-PTX to four breast cancer patients considered clinically to have contraindications to PTX or DOC. Two of the patients had previously experienced HSRs to PTX or DOC and the other two patients had contraindications to steroids as a premedication for HSRs, since one patient suffered from diabetes and the other was a carrier of the hepatitis B virus. All 4 patients were safely administered nab-PTX. In conclusion, administration of nab-PTX appears to be effective for patients that have previously experienced HSRs to other taxanes or in those with contraindications to steroids.

## Introduction

Taxanes, including paclitaxel (PTX) and docetaxel (DOC), are among the most active and widely used classes of cytotoxic agents for breast cancer treatment (1,2). Taxanes are poorly soluble in water due to their hydrophobic properties and thus, require solvents, including polyoxyethylated castor oil and ethanol (3). The use of these solvents has been associated with toxic responses, including hypersensitivity reactions (HSRs) and prolonged sensory neuropathy (3). Nanoparticle albumin-bound PTX (nab-PTX) is a novel formulation of PTX that allows reconstitution of this agent with a saline solution instead of solvents and administration without premedication for HSRs (4,5). The aim of the present study was to report the safety of nab-PTX, as a result of the administration of the formulation to 4 breast cancer patients with contraindications to PTX or DOC. Written informed consent was obtained from the patients.

## Case reports

### Case 1

In May 2004, a 43-year-old female with right-sided breast cancer underwent a partial resection of the breast (Bp) and axillary lymph node dissection (Ax). The patient was of pathological stage IIB [T2N1M0; estrogen receptor (ER)-positive, progesterone receptor (PR)-positive and human epidermal growth factor receptor 2 (HER2)-negative]. The patient was treated with adjuvant therapy consisting of 4 cycles of oral cyclophosphamide (100 mg/m^2^ daily on days 1–14), doxorubicin (30 mg/m^2^ on days 1 and 8) and 5-fluorouracil (500 mg/m^2^ on days 1 and 8) every 4 weeks and sequential endocrine therapy with tamoxifen (20 mg/day). At 4-years post-surgery, multiple bone metastases were detected. The patient was initially treated with endocrine therapy (2.5 mg/day letrozole followed by 25 mg/day exemestane) and zoledronic acid (4 mg every 3–4 weeks). After 21 months, the patient exhibited new lesions in the liver and the endocrine therapy was adjusted to chemotherapy with PTX (175 mg/m^2^ on day 1) and gemcitabine (1,250 mg/m^2^ on days 1 and 8) every three weeks.

**Figure 1:**
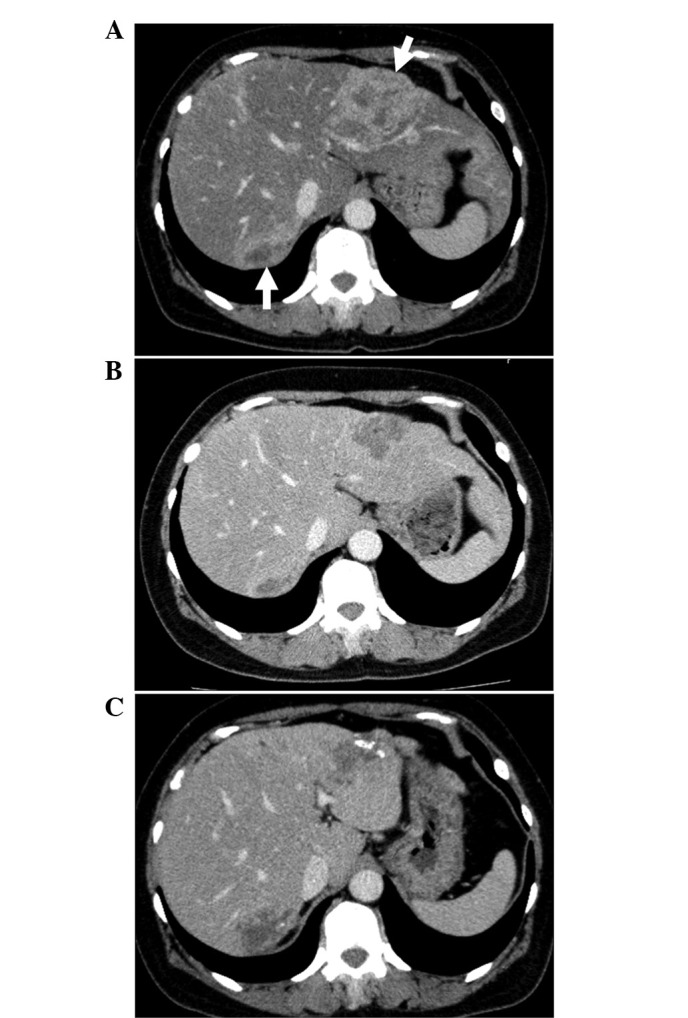
(A) Abdominal CT scan of a 43-year-old female with right-sided breast cancer showed multiple liver metastases, as indicated by the arrow. (B) CT following 4 cycles of therapy with nab-PTX showed stable disease, however, (C) following 11 cycles, disease progression in the liver was observed. CT, computed tomography; nab-PTX, nanoparticle albumin-bound paclitaxel.

PTX was administered via a 180-min infusion following premedication consisting of corticosteroid (dexamethasone, 20 mg/body) and antihistamines (ranitidine and diphenhydramine, both 50 mg/body). However, the patient developed dyspnea and flushing 10 mins after the initiation of intravenous administration. The symptoms were considered to be due to an HSR to PTX and the infusion was terminated. After 30 mins, the symptoms improved and, subsequently, the regimen was adjusted to capecitabine. After six months, the individual exhibited progressive disease in the liver (Fig. 1) and the regimen was adjusted to nab-PTX (260 mg/m^2^) every three weeks for 30 mins, with premedication of dexamethasone (8 mg) only. No HSRs were exhibited and the nab-PTX treatment was continued. Abdominal computed tomography (CT) was performed following 4 cycles of therapy and the patient exhibited a stable disease (SD) state. However, following 11 cycles of treatment, the patient experienced disease progression; therefore, nab-PTX treatment was discontinued (Fig. 1). During nab-PTX treatment, the patient exhibited no HSRs and sensory neuropathy and neutropenia were recorded as grade 1. Following nab-PTX treatment, the patient had three other chemotherapy treatments for seven months as follows: i) Six cycles of 1.4 mg/m^2^ eribulin mesylate on days 1 and 8 every three weeks; ii) four cycles of FEC (100 mg/m^2^ epirubicin, 500 mg/m^2^ 5-fluorouracil and 500 mg/m^2^ cyclophosphamide) every three weeks; and iii) one cycle of 1,200 mg/m^2^ gemcitabine and 25 mg/m^2^ vinorelbine on days 1 and 8 every three weeks. The outcome of the patient was good.

### Case 2

**Figure 2:**
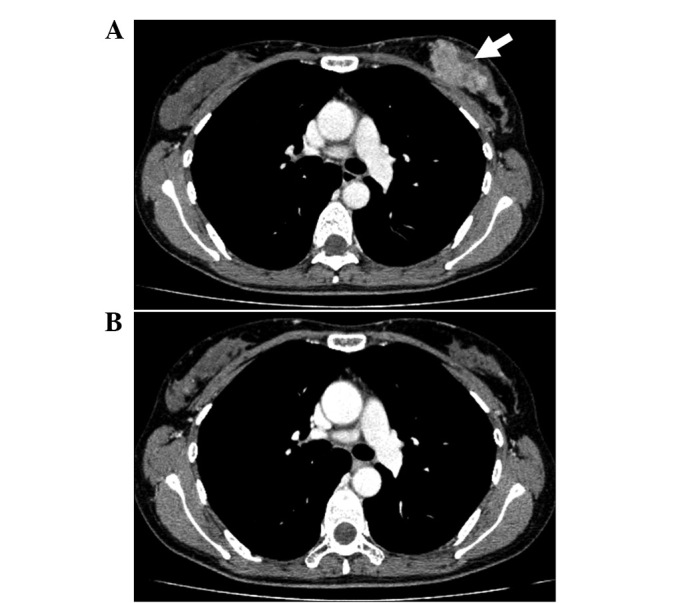
(A) Chest CT scan of a 36-year-old female showed left-sided breast cancer, as indicated by the arrow. (B) Following PST with 4 cycles of FEC, 1 cycle of DOC and 3 cycles of nab-PTX, the patient showed a cCR. CT, computed tomography; PST, pre-operative systemic therapy; nab-PTX, nanoparticle albumin-bound paclitaxel; FEC, 5-fluorouracil, epirubicin and cyclophosphamide; DOC, docetaxel; cCR, clinically complete response.

In May 2012, a 36-year-old female was diagnosed with left-sided breast cancer (stage IIIC; T3N3bM0; ER-positive, PR-positive and HER2-negative). The patient began pre-operative systemic therapy (PST) with 4 cycles of 5-fluorouracil (500 mg/m^2^), epirubicin (100 mg/m^2^) and cyclophosphamide (500 mg/m^2^) every 3 weeks, followed by 4 cycles of DOC (75 mg/m^2^) every 3 weeks. Dexamethasone (20 mg/body) was used as premedication for DOC. Although the patient received the initial cycle of DOC without developing an HSR, dyspnea and nausea developed 5 mins after administration of the second DOC. These symptoms were considered to be due to an HSR to DOC and the infusion was terminated. After 1 h, the symptoms improved and, subsequently, the regimen was adjusted to treatment with nab-PTX. The patient was administered nab-PTX (260 mg/m^2^) for 30 mins every 3 weeks, with premedication consisting of dexamethasone (8 mg) only. The patient exhibited no HSR and continued nab-PTX for 3 cycles. During the nab-PTX treatment, the patient exhibited no HSRs and sensory neuropathy and neutropenia were recorded as grade 1. Following PST, the patient experienced a clinically complete response (cCR; Fig. 2) and underwent a Bp and Ax. The patient had no recurrence.

### Case 3

In December 2000, a 45-year-old female with right-sided breast cancer underwent a total mastectomy and Ax. The patient was of pathological stage IIIB (T4bN2M0; ER-positive, PR-positive and HER2-negative). The patient was treated with 6 cycles of adjuvant chemotherapy with oral cyclophosphamide (100 mg/m^2^ daily on days 1–14), methotrexate (40 mg/m^2^ on days 1 and 8) and 5-fluorouracil (500 mg/m^2^ on days 1 and 8) every 4 weeks. At 9-years post-surgery, multiple lung metastases were detected and the patient was initially treated with anastrozole (1.0 mg/day). After 21 months, progressive disease was identified in the lung and FEC treatment (500 mg/m^2^ 5-fluorouracil, 100 mg/m^2^ epirubicin and 500 mg/m^2^ cyclophosphamide) was initiated.

**Figure 3:**
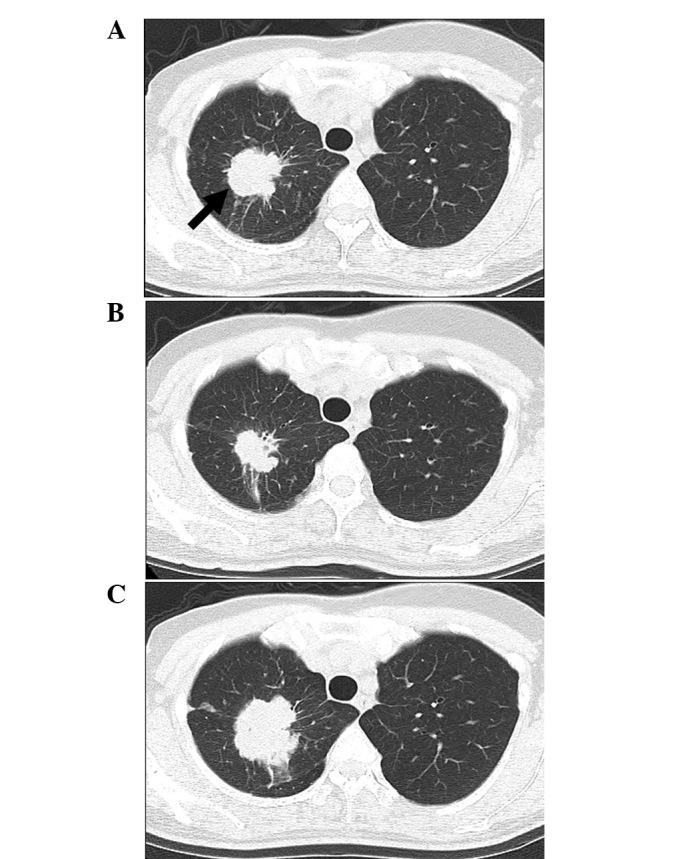
(A) CT scan of a 45-year-old female with right-sided breast cancer showed a lung metastasis, as indicated by the arrow. (B) CT following 4 cycles of therapy with nab-PTX showed a partial response, however, (C) following 15 cycles, disease progression was evident in the lung. CT, computed tomography; nab-PTX, nanoparticle albumin-bound paclitaxel.

The patient had a past medical history of diabetes and their HbA1c was controlled to <7% by metformin hydrochloride (2,250 mg/day). Following FEC initiation, the patient’s fasting blood sugar increased to >250 mg/dl and insulin glargine (6 U/day) was prescribed. However, the patient’s HbA1c continued to increase to 8.7% and the individual developed a thirst and general fatigue. The symptoms appeared to be associated with the dexamethasone, which was being used as a premedication for FEC. Therefore, the regimen was adjusted to nab-PTX (260 mg/m^2^) every 3 weeks. In January 2012, the patient was administered with nab-PTX for 30 mins without dexamethasone. No HSRs were exhibited and the nab-PTX treatment was continued for 15 cycles. The patient’s thirst and general fatigue improved, and four months after the adjustment to nab-PTX, the patient’s HbA1c also improved. A chest CT following 4 cycles of therapy showed a partial response of the lung metastases. However, following 15 cycles of therapy, the patient experienced disease progression; therefore, an-PTX treatment was discontinued (Fig. 3). During the nab-PTX treatment, the patient exhibited no HSRs and grade 2 sensory neuropathy was recorded. Following nab-PTX treatment, the patient was administered with capecitabine (2,400 mg/day for 21 days every 4 weeks) for 7 months until now.

### Case 4

In November 2012, a 47-year-old female with left-sided breast cancer underwent a Bp and Ax. The patient was of pathological stage IIIA (T2N2M0; ER-positive, PR-positive and HER2-negative). Although adjuvant chemotherapy with 4 cycles of FEC (500 mg/m^2^ 5-fluorouracil, 100 mg/m^2^ epirubicin and 500 mg/m^2^ cyclophosphamide) followed by 4 cycles of DOC (75 mg/m^2^) was initially prescribed, nab-PTX (260 mg/m^2^) without dexamethasone was selected since the patient was positive for the hepatitis B virus (HBV) antigen. Therefore, it was necessary to use entecavir hydrate and not a steroid as prophylaxis to avoid HBV reactivation. The patient completed the adjuvant chemotherapy, with no HSRs or HBV reactivation.

## Discussion

To ensure the safety of taxane treatment, PTX and DOC require solvents, including Cremophor EL and ethanol or polysorbate 80, and premedication with corticosteroids to reduce the risk of HSRs associated with these solvents (3,6). By contrast, nab-PTX may be administered without corticosteroids due to its novel formulation with albumin that allows reconstitution of nab-PTX with a saline solution instead of solvents (3).

A phase III trial previously compared the administration of nab-PTX (260 mg/m^2^) with PTX (175 mg/m^2^) every three weeks in patients with metastatic breast cancer (MBC). Nab-PTX demonstrated a significantly improved overall response rate and longer time to progression compared with PTX (5). In addition, the incidence of grade 4 neutropenia was significantly lower for nab-PTX compared with PTX. However, nab-PTX was associated with a higher rate of grade 3 sensory neuropathy compared with PTX. The incidence of HSRs in the trial was low for the two arms of the study (nab-PTX and PTX), however, patients receiving PTX were premedicated, whereas those receiving nab-PTX were not. A phase II trial performed in 2009 reported that patients with MBC administered with weekly nab-PTX (100 or 150 mg/m^2^ during the first 3/4 weeks) demonstrated a higher overall response rate and median progression-free survival compared with those administered with DOC (100 mg/m^2^ every three weeks) (7). In addition, a phase III trial in 2012 compared the administration of weekly nab-PTX (150 mg/m^2^) to that of weekly PTX (90 mg/m^2^) with bevacizumab, as first-line therapies for MBC. However, nab-PTX was not shown to be superior to PTX for progression-free survival (8). Therefore, it has been unclear which patients are likely to benefit from nab-PTX treatment.

The present study reports two patients who experienced HSRs associated with PTX and DOC and were subsequently administered nab-PTX, which did not result in an HSR. One patient (Case 1) was treated with nab-PTX in a metastatic setting and was able to continue treatment for 11 cycles with an overall response of a SD state. The other patient (case 2) was treated with PST and achieved cCR. Adverse reactions of the two patients were recorded as grade 1. Therefore, nab-PTX appears to be safe and effective for these patients. When HSRs to taxanes occur, the administration of the taxane is usually terminated. However, taxanes are among the most effective cytotoxic agents for breast cancer treatment. Therefore, we hypothesize that even if a patient has experienced HSRs to other taxanes, nab-PTX should be selected as a treatment, rather than withdrawing from the use of taxanes completely.

In addition, the current study reported two cases with contraindications to steroids. One was a patient with diabetes (case 3) who needed to avoid the poor glycemic control associated with steroid administration. Following treatment with nab-PTX, the patient exhibited no HSRs and good glycemic control. Nab-PTX was continued for 15 cycles and the highest overall response was a partial response. The other patient (case 4) was a carrier for HBV and, therefore, HBV reactivation had to be prevented. The patient completed adjuvant chemotherapy without any HSRs or HBV reactivation. The adverse reactions of the two patients were recorded as grade 1 and 2. Since nab-PTX does not require concomitant steroid treatment, this agent must be selected for patients with clinical contraindications to steroids.

In conclusion, nab-PTX administration appears to be safe and effective for patients who have previously experienced HSRs to other taxanes or in those who have contraindications to steroids.
